# Rumen Microbiome from Steers Differing in Feed Efficiency

**DOI:** 10.1371/journal.pone.0129174

**Published:** 2015-06-01

**Authors:** Phillip R. Myer, Timothy P. L. Smith, James E. Wells, Larry A. Kuehn, Harvey C. Freetly

**Affiliations:** USDA, ARS, U.S. Meat Animal Research Center, Clay Center, Nebraska, United States of America; Agriculture and Agri-Food Canada, CANADA

## Abstract

The cattle rumen has a diverse microbial ecosystem that is essential for the host to digest plant material. Extremes in body weight (BW) gain in mice and humans have been associated with different intestinal microbial populations. The objective of this study was to characterize the microbiome of the cattle rumen among steers differing in feed efficiency. Two contemporary groups of steers (*n*=148 and *n*=197) were fed a ration (dry matter basis) of 57.35% dry-rolled corn, 30% wet distillers grain with solubles, 8% alfalfa hay, 4.25% supplement, and 0.4% urea for 63 days. Individual feed intake (FI) and BW gain were determined. Within contemporary group, the four steers within each Cartesian quadrant were sampled (*n*=16/group) from the bivariate distribution of average daily BW gain and average daily FI. Bacterial 16S rRNA gene amplicons were sequenced from the harvested bovine rumen fluid samples using next-generation sequencing technology. No significant changes in diversity or richness were indicated, and UniFrac principal coordinate analysis did not show any separation of microbial communities within the rumen. However, the abundances of relative microbial populations and operational taxonomic units did reveal significant differences with reference to feed efficiency groups. Bacteroidetes and Firmicutes were the dominant phyla in all ruminal groups, with significant population shifts in relevant ruminal taxa, including phyla Firmicutes and Lentisphaerae, as well as genera *Succiniclasticum*, *Lactobacillus*, *Ruminococcus*, and *Prevotella*. This study suggests the involvement of the rumen microbiome as a component influencing the efficiency of weight gain at the 16S level, which can be utilized to better understand variations in microbial ecology as well as host factors that will improve feed efficiency.

## Introduction

The bovine gastrointestinal (GI) tract is a complex system that is responsible for animal nutrient uptake and overall health. Rumen function and the microbiology of culturable rumen microorganisms has been well-studied, but it has only been since the advent of next-generation sequencing technology in the last decade that research on total microbial diversity, microbial community function, and their effects on the host could be quantitatively examined at a higher resolution. These new technologies have enabled a thorough examination of the host microbiota, independent of culture-based methods.

The gastrointestinal tract is host to a diverse microbial ecosystem that can vary depending on both host genetic and environmental factors [1]. Studies have shown that even minor shifts in these populations can have a tremendous impact on livestock nutrition and productivity [2–4]. Changes in the composition and diversity of the ruminal microbiota have been linked with diet and age [5]. Extremes in body weight (BW) gain in mice and humans have been associated with different intestinal microbial populations [6–7]. However, the majority of research in cattle has focused on the microbial responses to changes in external parameters, such as diet influences, and management practices [8–9]. Little has been done to examine the effect of differing microbial populations on host phenotypes, specifically related to performance and feed efficiency. Furthermore, many of these studies lack adequate sample size; examining only a few subjects. With advances in sequencing technology, researchers can examine these relationships with greater depth and coverage than has previously been reported.

Feed costs remain the largest variable cost in beef production [10]. Optimizing feed efficiency in cattle has long been an effort devoted to host genetics, management, and diet. Yet, data supports possible microbial interactions within the host, influencing a multitude of processes pertaining to digestion and the host GI tract. We hypothesize that variable microbial populations have differential abilities to degrade feedstuffs in the rumen into nutrients available for absorption, as a possible route to impacting feed efficiency. This study aimed to characterize the microbiome, specifically the bacterial community, of the cattle rumen among steers differing in feed intake and growth, in order to assess the association of the ruminal microbial community profile with variation in bovine feed efficiency.

## Materials and Methods

### Ethics Statement

This experiment was approved by the U.S. Meat Animal Research Center Animal Care and Use Committee.

### Experimental design and rumen sampling

**Fig 1:**
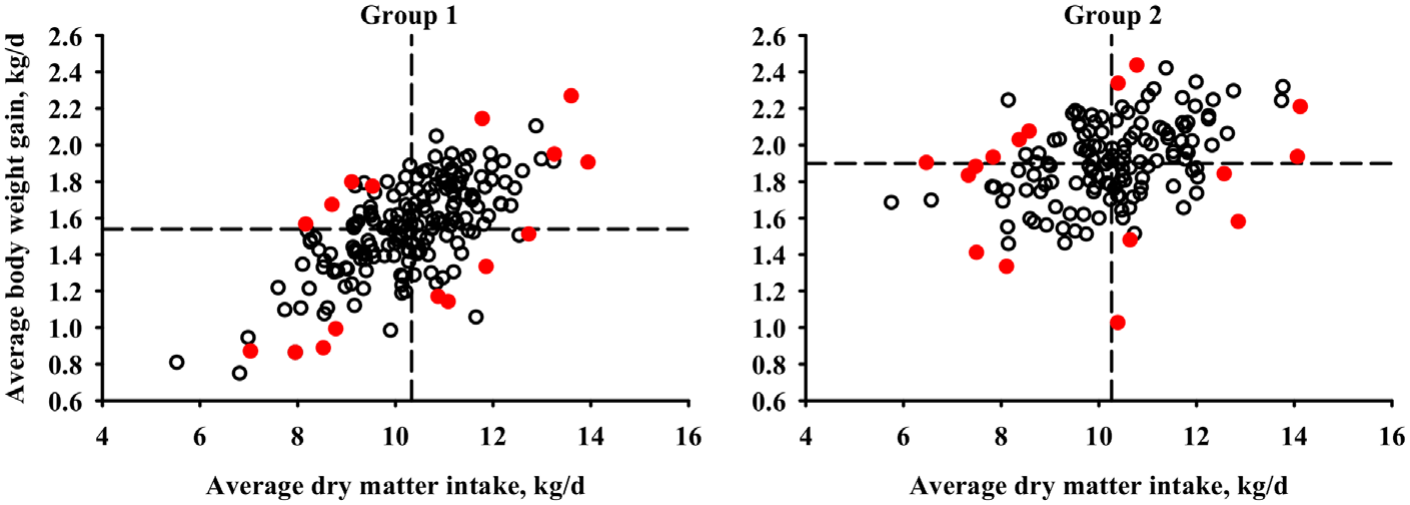
Feed Efficiency Sampling Model. A 2x2 factorial design consisting of high and low average daily feed intake (ADFI), and high and low average daily BW gain (ADG). Within a contemporary group, ADG was regressed on ADFI. Steers within each Cartesian quadrant were sampled, as represented by red dots (n = 16/group). Contemporary group 1(n = 197) ADFI was 10.33 ± 0.09 kg/d, and ADG was 1.54 ± 0.02 kg/d. Contemporary group 2 (n = 148) ADFI was 10.26 ± 0.12 kg/d, ADG was 1.90 ± 0.02 kg/d.

Steers selected for this study came from a populations of cattle being developed to have a high percentage of the following breeds: Angus, Beefmaster, Brahman, Brangus, Braunvieh, Charolais, Chiangus, Gelbvieh, Hereford, Limousin, Maine Anjou, Red Angus, Salers, Santa Gertrudis, Shorthorn, Simmental, South Devon, and Tarentaise. Each year heifers and cows were artificially inseminated with semen from prominent industry bulls of their dominant breed. This program resulted in offspring ranging from 50% to 75% of the same breed as their sire with the exception of Angus and Hereford which ranged from 50% to 100% of the same breed as their sire. Individual feed intake was measured using an Insentec feeding system (Marknesse, The Netherlands). Steers were fed a ration (dry matter basis) of 57.35% dry-rolled corn, 30% wet distillers grain with solubles, 8% alfalfa hay, 4.25% supplement (containing 0.772 g/kg monensin), and 0.4% urea. Feed intake and BW gain were measured over a 63 day period [11]. Steers were selected from two contemporary groups. Group 1 (*n* = 148) were spring-born calves that were 371 ± 1 d of age and weighed 522 ± 4 kg at the start of the feed intake measurement. Group 2 (*n* = 197) were fall-born calves that were 343 ± 1 d of age and weighed 448 ± 4 kg at the start of the feed intake measurement. At the end of each feeding period, average daily BW gain (ADG) was regressed on average daily dry matter intake (ADFI). The four steers within each Cartesian quadrant were sampled (*n* = 16/group) from the bivariate distribution of ADG and ADFI. The result was a 2 X 2 factorial design consisting of high and low ADFI, and high and low ADG (Fig 1). At the end of the feeding period, steers were harvested, and rumen fluid was sampled and strained through 4 layers of cheesecloth. The 2 feeding studies yielded 32 animals for analysis. Due to the high-concentrate diet and whole-sample straining, rumen fluid samples were used for this study. Samples were individually stored in buffered peptone water (BPW, pH 7.0) + 15% glycerol stock for processing and kept at -70°C for long-term storage post-processing.

### DNA extraction, amplification and sequencing

DNA was extracted from rumen samples using a repeated bead beating plus column (RBB+C) method [12]. Briefly, 0.3g of sample was centrifuged for 5 min at 16,000*× g* to pellet solids including bacterial cells, then resuspended in 0.2 mL TE (Tris-EDTA, pH 8.0) buffer. Cell lysis was achieved by bead beating 0.15 g of the resuspended sample in ZR BashingBead Lysis Tubes (Zymo Research Corp, Santa Ana, CA, USA) using the TissueLyser II system (Qiagen, Hilden, Germany) for 3 min at 21Hz, in the presence of 4% (w/v) sodium dodecyl sulfate (SDS), 500 mM NaCl, and 50 mM EDTA. After mechanical and chemical lysis, ammonium acetate was used to precipitate and remove the impurities and the SDS, along with isopropanol precipitation for the recovery of the nucleic acids. RNA and proteins were removed or degraded using RNase and proteinase K, followed by the use of QIAamp columns from the Qiagen DNA Stool Mini Kit (Qiagen, Hilden, Germany). Genomic DNA concentration was determined using a Nanodrop 1000 spectrophotometer (ThermoScientific, Wilmington, DE, USA).

Amplicon library preparation was performed by PCR amplification of the V1–V3 region of 16S rRNA gene, using modified universal primers 27F (5'- Adapter / Index / AGAGTTTGATCCTGGCTCAG) and 519R (5' Adapter / Index / GTATTACCGCGGCTGCTG) including TruSeq adapter sequences and indices, as well as AccuPrime Taq high fidelity DNA Polymerase (Life Technologies, Carlsbad, CA). Amplification consisted of 20 cycles, with an annealing temperature of 58°C. Products were purified using AmPure bead purification (Agencourt, Beverly, MA) and all libraries were quantified by the PicoGreen dsDNA quantitation kit (Invitrogen, Carlsbad, CA) and by real-time PCR on the LightCycler 480 system (Roche, Mannheim, Germany). The PCR amplicon libraries were sequenced using the 2x300, v3 600-cycle kit and the Illumina MiSeq sequencing platform (Illumina, San Diego, CA).

### Sequence read processing and analysis

All sequences were processed using the QIIME-1.8.0 software package. Paired reads were joined using fastq-join [13] and filtered for quality (≥Q25) using the Galaxy server [14]. Sequences that contained read lengths shorter than 400 bp were removed and adapters/index sequences were trimmed. Chimeric sequences were checked using ChimeraSlayer [15]. All cleaned sequences were classified into taxa using the Greengenes 16S rRNA Gene Database [16]. Operational taxonomic units (OTUs) were calculated using the uclust program at 0.03 dissimilarity [17]. After calculating richness for each quadrant, singletons were removed from further diversity analyses. Based on rarefaction curves, the number of OTUs was normalized via random subsampling of 25,000 sequences from each rumen sample. A phylogenic tree was built with FastTree [18] to determine alpha and beta diversity metrics.

### Statistical analysis

The mean abundances (n = 8) of data metrics and each taxon were compared among the feed efficiency groups using a model of contemporary group and Cartesian quadrant ((high ADG, high ADFI (ADG_High_-ADFI_High_); high ADG, low ADFI (ADG_High_-ADFI_Low_); low ADG, low ADFI (ADG_Low_-ADFI_Low_); low ADG, high ADFI (ADG_Low_-ADFI_High_)) as fixed effects. The Benjamini–Hochberg method was used for multiple-testing correction [19]. Multiple-testing corrections were made for the number of phyla, the number of OTU groups, and other classified taxa groups. Significant difference was determined at P < 0.05. Linear contrasts were then applied to quadrant to separate whether microbial populations varied by low vs. high ADG, low vs. high ADFI, or their interaction (P < 0.01). Principal coordinates analysis (PCoA) was performed using weighted and unweighted UniFrac analyses [20].

## Results

### Diversity of Ruminal Bacterial Communities

After quality control, chimera detection, and removal, the sampled ruminal contents of 32 steers, grouped into 4 feed efficiency phenotypes yielded a total of 5,565,909 cleaned reads with an average read length of 500 bp. The total cleaned reads represented individual samples ranging from 25,094 to 888,867 reads. OTUs were defined as a read sharing ≥97% nucleotide sequence identity. From the total sequences, 68,655 OTUs were detected with an average of 17,164±3619 OTUs per quadrant. From each Cartesian quadrant, data ranged from 13,304–21,971 OTUs, with the greatest number of OTUs stemming from the ADG_High_-ADFI_High_ group and the fewest from the ADG_Low_-ADFI_High_ group. The quadrants representing ADG_High_-ADFI_Low_ and ADG_Low_-ADFI_Low_ yielded 17,337 and 16,043 OTUs, respectively. The number of singletons accounted for approximately 38% of all OTUs. Coverage ranged from 98.31 to 99.56% as determined by Good's coverage estimator. Richness (Shannon) was slightly reduced, but not significant in the ADG_High_-ADFI_High_ group at 6.32 compared to the other 3 groups (6.89–7.11).

In order to normalize individual samples for comparison among phenotype classes, each sample OTU table was rarefied to 25,000 reads, which was based upon optimizing both minimum sample read output and sample rarefaction curves. Each normalized sample was then analyzed and compared using the sample means within each quadrant. The normalized sequence reads were examined via bacterial diversity (Shannon), richness (Chao-1), and coverage (Good's coverage estimator). The average number of OTUs from the normalized samples did not differ (*P* > 0.05) among the 4 feed efficiency groups, which had an average of 1098±382 OTUs per group (Table 1). The data representing richness similarly did not indicate any significance (*P* > 0.05) among the number of OTUs, which ranged from 1298–1601. Though some variability was observed, the diversity indices for each group were not significantly different. Each group was adequately covered, up to 98.84% in the ADG_Low_-ADFI_High_ group and as low as 98.37% in the ADG_High_-ADFI_High_ group.

**Table 1:** Diversity statistics among reads from grouped samples.

Feed Efficiency Group	Sampling Type	No. of Sequences	No. of OTUs^1^	Chao1	Shannon Diversity Index	Good's Coverage (%)
ADG_High -_ADFI_High_ ^2^	Subsampled Reads^3^	25,000	1069 ± 309^a^	1601 ± 485^a^	6.32 ± 1.21^a^	98.37 ± 0.78
ADG_High -_ADFI_Low_ ^2^	Subsampled Reads^3^	25,000	1078 ± 479^a^	1380 ± 424^a^	7.11 ± 0.93^a^	98.77 ± 1.00
ADG_Low -_ADFI_Low_ ^2^	Subsampled Reads^3^	25,000	1172 ± 394^a^	1438 ± 468^a^	7.06 ± 0.96^a^	98.82 ± 0.97
ADG_Low -_ADFI_High_ ^2^	Subsampled Reads^3^	25,000	1072 ± 345^a^	1298 ± 349^a^	6.89 ± 0.28^a^	98.84 ± 0.83

^abc^Within a column, means for the individual subsamples having different superscripts differ (*P*<0.05.)

^1^OTUs represents Operational Taxonomic Units.

^2^ *n* = 8 among groups.

^3^Means among the groups were compared using ANOVA and the Tukey's Test.

**Fig 2:**
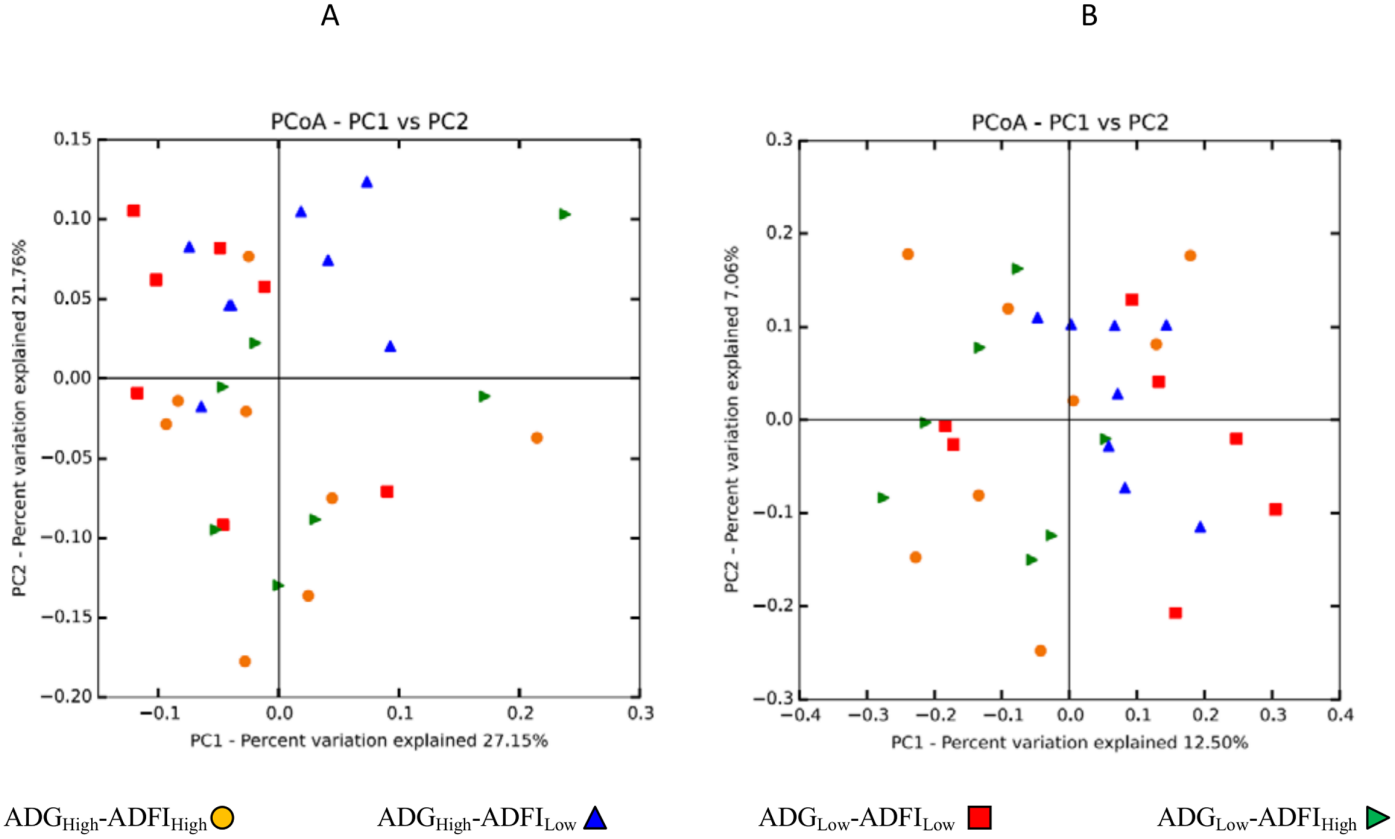
UniFrac principal coordinates analysis (PCoA) displaying correlations among the bacterial communities of the 4 groups. A) Weighted PCoA analyzed from rarefied subsets of 25,000 sequences from each sample. B) Unweighted PCoA analyzed from rarefied subsets of 25,000 sequences from each sample. *n* = 8, represented by differing symbols.

The data was then reduced to an OTU-centric method, principal coordinates analysis (PCoA), to determine any separation into sample clusters. This was achieved by applying the phylogeny-based method, UniFrac, to the data. This method is a β-diversity measure that takes the phylogenic divergence between the OTUs into account aiding in identification of differences among microbial communities [21]. In both the weighted (quantitative) and unweighted (qualitative) UniFrac distances [22], there was no separation into clusters observed in the PCoA (Fig 2).

### Taxonomic and OTU Composition

The 5,565,909 cleaned reads were classified by the Greengenes 16S rRNA Gene Database [16] into 24 phyla, 48 classes, 89 orders, 173 families, and 317 genera. Bacteroidetes (53–63%) and Firmicutes (23–33%) dominated the phylum dataset within each subsampled group (in terms of percent of the total reads; Table 2), similar to previous studies of rumen microflora [23]. The remaining phyla represent less than 1% of the remaining cleaned reads. Proteobacteria, Tenericutes, Cyanobacteria, and Spirochaetes accounted for the less dominant phyla (greater than 0.5%). Additionally, about 9% of the reads could not be classified into a known phylum.

**Table 2:** Relative abundance of taxa in the four groups.

		Percentage of Total Sequences^1^ ^,^ ^2^					
							No. of Steers with
Phylum	Sub Classification	ADG_High -_ADFI_High_	ADG_High -_ADFI_Low_	ADG_Low -_ADFI_Low_	ADG_Low -_ADFI_High_	*P*-value^3^	Detectable Taxon^4^
Bacteroidetes		63.826 (4.20)	53.225 (3.91)	61.847 (3.92)	57.253 (4.20)	0.2999	32
	*Prevotella*	57.161 (4.94)	45.367 (4.61)	49.557 (4.61)	47.635 (4.94)	0.3882	32
	Order Bacteroidales	1.566 (0.88)	3.031 (0.83)	2.265 (0.83)	2.317 (0.88)	0.6540	32
	Order Bacteroidales	2.148 (0.64)	2.303 (0.60)	3.344 (0.60)	3.586 (0.64)	0.3555	32
	Order Bacteroidales; Family RF16	0.573 (0.50)	1.420 (0.46)	0.462 (0.46)	0.639 (0.50)	0.5319	31
	Family Paraprevotellaceae; Genus *YRC22*	0.291 (0.10)	0.162 (0.09)	0.150 (0.9)	0.326 (0.10)	0.6213	32
Firmicutes		25.242 (2.64)	33.434 (2.47)	22.998 (2.47)	28.991 (2.64)	**0.0364**	32
	*Dialister*	2.593 (0.55)	4.055 (0.51)	0.399 (0.51)	1.039 (0.55)	0.0002	29
	*Succiniclasticum*	2.223 (0.70)	1.711 (0.66)	2.421 (0.66)	4.020 (0.70)	0.1400	32
	*Lactobacillus*	0.584 (0.09)	0.461 (0.09)	0.212 (0.09)	0.331 (0.09)	**0.0419**	32
	*Ruminococcus*	1.092 (0.64)	2.565 (0.61)	2.322 (0.61)	2.273 (0.64)	0.1900	32
	*Butyrivibrio*	1.033 (0.33)	1.133 (0.31)	0.578 (0.31)	0.716 (0.33)	0.5174	32
	*Lachnospira*	0.272 (0.13)	0.244 (0.12)	0.109 (0.12)	0.296 (0.13)	0.7779	27
	*Megasphaera*	0.433 (0.15)	0.467 (0.14)	0.291 (0.14)	0.105 (0.13)	0.4729	29
	*Mitsuokella*	1.204 (0.32)	0.984 (0.30)	0.990 (0.30)	0.610 (0.32)	0.6277	32
	*Moryella*	0.142 (0.14)	0.185 (0.13)	0.405 (0.13)	0.538 (0.14)	0.1930	31
	*Shuttleworthia*	0.496 (0.27)	0.397 (0.25)	0.480 (0.25)	0.388 (0.27)	0.9666	29
	*Bulleidia*	0.363 (0.08)	0.284 (0.07)	0.183 (0.07)	0.239 (0.08)	0.5118	32
	*Acidaminococcus*	0.322 (0.07)	0.254 (0.07)	0.050 (0.07)	0.101 (0.07)	**0.0306**	28
	*Anaerovibrio*	0.004 (0.02)	0.012 (0.02)	0.085 (0.02)	0.028 (0.02)	**0.0291**	29
	Family Lachnospiraceae	5.113 (0.67)	4.847 (0.63)	2.671 (0.63)	2.808 (0.67)	**0.0197**	32
	Order Clostridiales	1.592 (0.54)	2.489 (0.51)	2.084 (0.51)	2.895 (0.54)	0.1949	32
	Family Ruminococcaceae	2.334 (1.33)	4.317 (0.24)	4.121 (1.24)	7.071 (1.33)	0.1071	32
	Family Lachnospiraceae	5.027 (0.67)	4.847 (0.63)	2.671 (0.63)	2.722 (0.67)	**0.0197**	32
	Family Veillonellaceae	2.692 (0.59)	4.254 (0.55)	1.671 (0.55)	1.281 (0.59)	**0.0058**	32
	Family Mogibacteriaceae	0.497 (0.28)	1.276 (0.26)	0.786 (0.26)	0.881 (0.28)	0.6684	32
	Family Lachnospiraceae	0.585 (0.38)	1.333 (0.36)	0.314 (0.36)	0.269 (0.38)	0.1619	31
	Family Erysipelotrichaceae; Genus *RFN20*	0.152 (0.10)	0.582 (0.09)	0.175 (0.09)	0.408 (0.10)	**0.0182**	30
Unassigned		7.906 (2.83)	9.783 (2.64)	11.464 (2.64)	10.560 (2.83)	0.7858	32
Proteobacteria		0.715 (0.25)	0.999 (0.23)	0.904 (0.23)	0.599 (0.25)	0.5829	32
	*Lysobacter*	4.318 x 10^–5^ (0.0003)	4.235 x 10^–20^ (0.0003)	1.270 x10^-19^ (0.0003)	0.001 (0.0003)	**0.0462**	29
	Family Helicobacteraceae	5.702 x 10^–5^ (0.0003)	0.001 (0.0003)	8.470 x 10^–20^ (0.0003)	5.701 x 10^–5^ (0.0003)	**0.0160**	28
Tenericutes		0.337 (0.38)	0.509 (0.35)	0.815 (0.35)	0.555 (0.38)	0.1691	32
Cyanobacteria		0.889 (0.28)	0.555 (0.26)	0.439 (0.26)	0.321 (0.28)	0.6253	32
Synergistetes		0.175 (0.09)	0.348 (0.09)	0.348 (0.09)	0.169 (0.09)	0.2501	32
Fibrobacteres		0.109 (0.04)	0.0739 (0.04)	0.061 (0.04)	0.171 (0.04)	0.4448	32
Actinobacteria		0.119 (0.16)	0.427 (0.15)	0.166 (0.15)	0.143 (0.16)	0.3870	32
	*Janibacter*	5.711 x 10^–5^ (0.0003)	3.388 x 10^–19^ (0.0003)	5.711 x 10^–5^ (0.0003)	0.001 (0.0003)	**0.0161**	29
	*Leucobacter*	2.153 x 10^–5^ (0.0005)	0.001 (0.0005)	3.176 x 10^–19^ (0.0005)	5.815 x 10^–4^ (0.0005)	**0.0215**	30
TM7		0.093 (0.04)	0.108 (0.04)	0.093 (0.04)	0.159 (0.04)	0.2702	31
Spirochaetes		0.405 (0.19)	0.252 (0.18)	0.542 (0.18)	0.230 (0.19)	0.6129	32
Chloroflexi		0.039 (0.18)	0.075 (0.17)	0.116 (0.17)	0.549 (0.19)	0.2498	32
SR1		0.010 (0.01)	0.0243 (0.01)	0.006 (0.01)	0.006 (0.01)	0.1148	21
Elusimicrobia		0.033 (0.03)	0.117 (0.03)	0.019 (0.03)	0.048 (0.03)	0.2764	27
Fusobacteria		0.008 (0.004)	0.003 (0.004)	0.005 (0.004)	0.001 (0.004)	0.6976	14
Lentisphaerae		0.013 (0.02)	0.008 (0.02)	0.052 (0.02)	0.093 (0.02)	**0.0339**	25
	Family Victivallaceae	0.011 (0.02)	0.008 (0.02)	0.049 (0.02)	0.090 (0.02)	**0.0358**	31
Planctomycetes		0.024 (0.04)	0.025 (0.03)	0.077 (0.03)	0.126 (0.04)	0.2593	28
Verrucomicrobia		0.019 (0.01)	0.012 (0.01)	0.027 (0.01)	0.011 (0.01)	0.8390	19

^1^Data is shown as LSMeans with standard errors in parentheses.

^2^ *n* = 8 among groups.

^3^Bold *P*-values indicate groups that differ (*P*<0.05).

^4^Percentage of total sequences for steers with nondetectable taxon were treated as 0.001%.

At the genus level, *Prevotella* was in greatest abundance at 45–57%, followed by *Dialister* (2.6–4.1%), *Succiniclasticum* (2.0–4.0%), *Ruminococcus* (1.0–2.5%), *Butyrvibrio*(0.5–1.1%), and *Mitsuokella* (0.6–1.2%), which represented genera at ≥1% of the total reads (Table 2). A number of taxa were not classified to the genus level, but were present in great abundance. Several abundances within the order of Bacteroidales were prevalent, as well as Clostridiales and the families Ruminococcaceae, Lachnospiraceae, and Veillonellaceae (Table 2). The remaining taxa were not listed and deemed non-detectable at abundances ≤ 0.001%.

**Fig 3:**
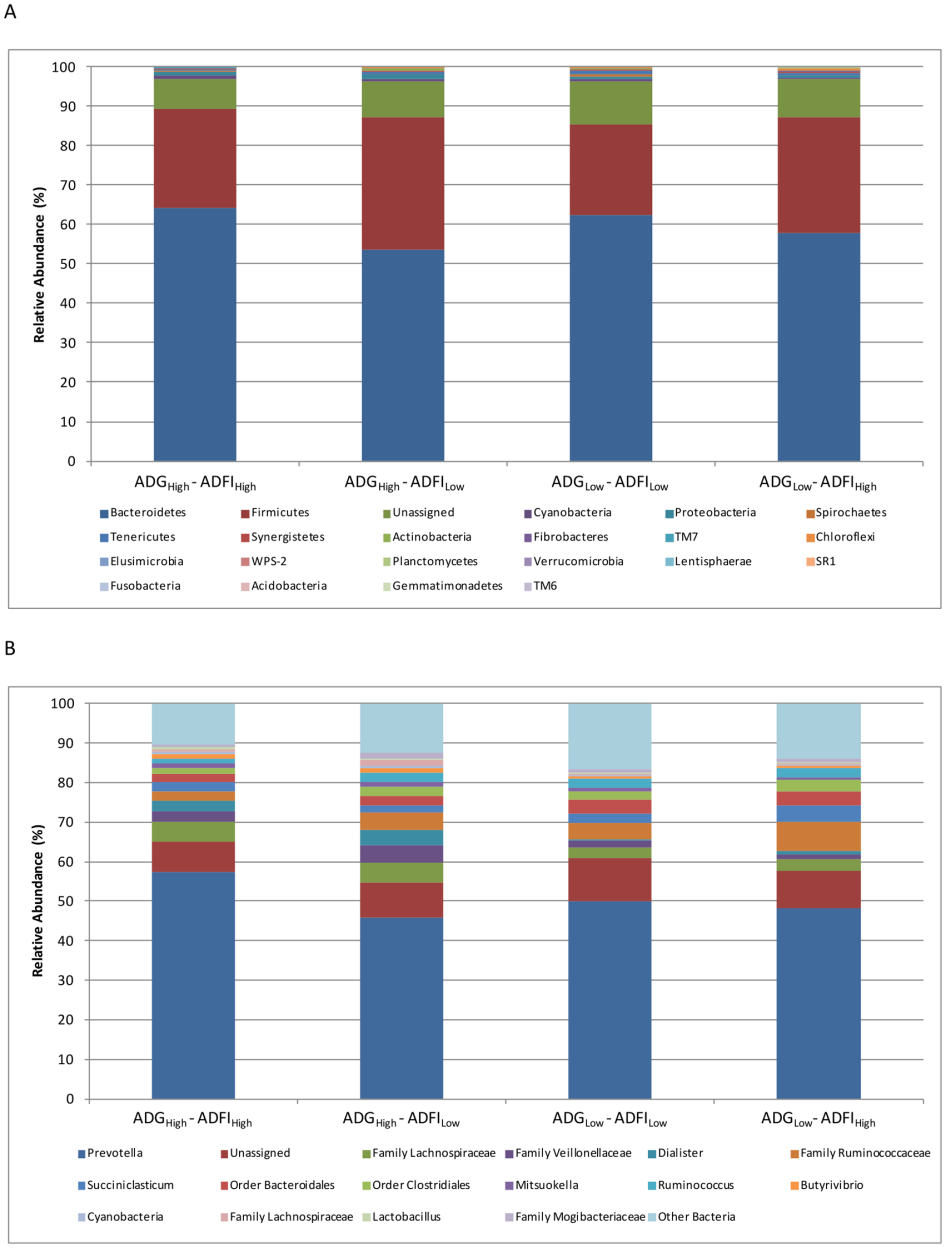
The taxonomic profiles for the relative phylum-level (A) and genus-level (B) abundance of each group classified by representation at >1% of total sequences. Taxonomic composition of the ruminal microbiota among the four groups was compared based on the relative abundance (reads of a taxon/total reads in a sample).

Analysis of the relative taxonomic abundance across all feed efficiency groups revealed differences among phyla and genera (Table 2 and Fig 3). The data were analyzed to reflect the mean of the relative abundance (reads of a taxon/total reads in a sample) in each feed efficiency group. The phyla Firmicutes (*P* = 0.0364) and Lentisphaerae (*P* = 0.0339), and genera *Dialister* (*P* = 0.0062), *Lactobacillus* (*P* = 0.0419), *Acidaminococcus* (*P =* 0.0306), *Anaerovibrio* (*P =* 0.0291) *Lysobacter* (*P =* 0.0462), *Janibacter* (*P =* 0.0161), *and Leucobacter* (*P =* 0.0215) demonstrated significant differences in abundance between the feed efficiency groups. Additionally, several lower orders of classification were significant between the groups, which included the families Lachnospiraceae (*P* = 0.0197), Veillonellaceae (*P* = 0.0058), and Helicobacteraceae (*P* = 0.0160). Examination of the taxonomic profiles for the relative phylum-level and genus-level abundances demonstrated changes between the efficiency groups. This was most evident within the Bacteroidetes, Firmicutes, and *Prevotella* identities (Fig 3). However, the high variability permitted limited determination of any visual trends within the profiles, specifically at lower abundances.

Using an OTU-centric approach, OTUs classified to *Prevotella* were the most dominant among the OTUs in the collective data (Table 3). Again, analysis across all feed efficiency groups revealed differences among OTUs, which included OTUs classified to the families Veillonellaceae (*P* = 0.0492) and Ruminococcaceae (*P* = 0.0241), and the genera *Prevotella* (*P* = 0.0319), *Lactobacillus* (*P =* 0.0484), *Dialister* (*P* = 0.0323), *Butyrivibrio* (*P =* 0.0391), *Succiniclasticum* (*P* = 0.0308), and *Ruminococcus (P =* 0.0255). OTUs listed were deemed non-detectable at abundances ≤ 0.001%.

**Table 3:** Relative abundance of OTUs in the four groups.

		Percentage of Total Sequences^1^ ^,^ ^2^					
		ADG_High_ -	ADG_High_ -	ADG_Low_ -	ADG_Low_ -		No. of Steers with
OTU ID	Classification	ADFI_High_	ADFI_Low_	ADFI_Low_	ADFI_High_	*P*-value^3^	Detectable Taxon^4^
denovo693	Unclassified Veillonellaceae	0.030 (0.007)	0.025 (0.007)	0.003 (0.007)	0.005 (0.007)	**0.0247**	23
denovo727	Unclassified Bacteroidales	0.012 (0.01)	0.011 (0.01)	0.001 (0.01)	0.082 (0.01)	**0.0284**	17
denovo924	Unclassified Veillonellaceae	0.065 (0.02)	0.109 (0.02)	0.026 (0.02)	0.023 (0.02)	**0.0409**	21
denovo1346	*Lactobacillus*	0.096 (0.01)	0.060 (0.01)	0.019 (0.01)	0.046 (0.01)	**0.0484**	28
denovo1446	Unclassified Veillonellaceae	0.005 (0.004)	0.017 (0.004)	0.004 (0.004)	0.003 (0.004)	**0.0409**	19
denovo1927	*Selenomonas ruminantium*	0.005 (0.001)	0.017 (0.001)	0.004 (0.001)	0.039 (0.001)	**0.0402**	22
denovo2844	Unclassified Ruminococcaceae	0.864 (0.43)	1.238 (0.40)	0.521 (0.40)	2.429 (0.43)	**0.0241**	29
denovo2901	*Mitsuokella*	0.748 (0.18)	0.588 (0.17)	0.540 (0.17)	0.381 (0.18)	0.5469	32
denovo2963	Unclassified Clostridiales	0.012 (0.02)	0.076 (0.02)	0.006 (0.02)	0.015 (0.02)	**0.0441**	18
denovo3084	*Succiniclasticum*	1.334 (0.59)	0.668 (0.55)	1.835 (0.55)	3.251 (0.59)	**0.0276**	30
denovo3724	*Dialister*	0.918 (0.28)	1.724 (0.27)	0.176 (0.27)	0.361 (0.28)	**0.0018**	23
denovo4476	Unclassified Bacteroidales	0.012 (0.007)	0.014 (0.007)	0.023 (0.007)	0.058 (0.007)	**0.0005**	27
denovo4580	*Selenomonas*	0.131 (0.02)	0.068 (0.02)	0.008 (0.02)	0.014 (0.02)	**0.0146**	19
denovo4857	Unclassified Coriobacteriaceae	0.016 (0.003)	0.004 (0.003)	0.004 (0.003)	0.0137 (0.003)	**0.0056**	23
denovo5361	*Bulleidia*	0.001 (0.002)	0.001 (0.002)	0.010 (0.002)	0.010 (0.002)	**0.0110**	17
denovo5654	Unclassified Veillonellaceae	0.022 (0.01)	0.072 (0.01)	0.025 (0.01)	0.017 (0.01)	**0.0146**	26
denovo6377	*Prevotella*	3.632 (0.99)	3.393 (0.93)	1.454 (0.93)	1.138 (0.99)	0.1655	26
denovo6994	Unclassified Mogibacteriaceae	0.005 (0.002)	0.003 (0.002)	0.002 (0.002)	0.013 (0.002)	**0.0276**	21
denovo7333	*Prevotella*	2.356 (0.52)	0.6975 (0.49)	0.3465 (0.49)	0.172 (0.52)	**0.0259**	26
denovo7855	*Prevotella*	3.402 (1.21)	3.981 (1.12)	3.910 (1.12)	5.688 (1.21)	0.4783	31
denovo8451	Unclassified Bacteroidales	1.295 (1.30)	0.468 (1.22)	4.428 (1.22)	0.773 (1.30)	0.0589	29
denovo8632	Unclassified Veillonellaceae	0.016 (0.008)	0.037 (0.007)	0.013 (0.007)	0.005 (0.008)	**0.0434**	22
denovo8823	Unclassified Cyanobacteria	0.219 (0.05)	0.019 (0.05)	0.009 (0.05)	0.0089 (0.05)	**0.0319**	23
denovo8962	*Prevotella*	0.266 (0.04)	0.181 (0.04)	0.053 (0.04)	0.072 (0.04)	**0.0154**	26
denovo9246	*Dialister*	0.554 (0.18)	0.716 (0.16)	0.027 (0.16)	0.200 (0.18)	**0.0323**	22
denovo9974	*Butyrivibrio*	0.062 (0.02)	0.110 (0.02)	0.008 (0.02)	0.010 (0.02)	**0.0391**	18
denovo10250	*Prevotella*	0.012 (0.002)	0.007 (0.002)	0.004 (0.002)	0.0001 (0.002)	**0.0215**	18
denovo10316	Unclassified Veillonellaceae	0.048 (0.02)	0.075 (0.02)	0.006 (0.02)	0.007 (0.02)	**0.0399**	22
denovo10446	Unclassified Veillonellaceae	0.134 (0.03)	0.212 (0.03)	0.058 (0.03)	0.068 (0.03)	**0.0194**	30
denovo10455	Unclassified Streptophyta	0.009 (0.001)	0.007 (0.001)	0.003 (0.001)	0.001 (0.001)	**0.0261**	17
denovo11609	*Succiniclasticum*	0.403 (0.24)	0.689 (0.23)	0.405 (0.23)	0.641 (0.24)	0.8619	29
denovo12086	*Prevotella*	0.124 (0.08)	0.526 (0.07)	0.056 (0.07)	0.078 (0.08)	**0.0003**	26
denovo12190	Unclassified Veillonellaceae	0.060 (0.04)	0.191 (0.04)	0.061 (0.04)	0.021 (0.04)	**0.0323**	30
denovo12367	*Ruminococcus*	0.001 (0.005)	0.008 (0.005)	0.025 (0.005)	0.004 (0.005)	**0.0255**	17
denovo12649	Unclassified Veillonellaceae	0.059 (0.06)	0.319 (0.06)	0.060 (0.06)	0.018 (0.06)	**0.0071**	25
denovo12680	*Succiniclasticum*	0.499 (0.11)	0.335 (0.11)	0.049 (0.11)	0.029 (0.11)	**0.0154**	23
denovo12685	Unclassified Veillonellaceae	0.034 (0.02)	0.128 (0.02)	0.028 (0.02)	0.008 (0.02)	**0.0113**	23
denovo12932	Unclassified Mogibacteriaceae	0.001 (0.003)	0.003 (0.002)	0.005 (0.002)	0.017 (0.003)	**0.0202**	19
denovo13008	Unclassified Veillonellaceae	0.065 (0.02)	0.098 (0.02)	0.013 (0.02)	0.009 (0.02)	**0.0342**	23
denovo13305	*Prevotella*	16.549 (3.43)	6.304 (3.20)	5.236 (3.20)	5.17 (3.43)	0.0802	32
denovo14434	Unclassified Veillonellaceae	0.006 (0.004)	0.030 (0.004)	0.009 (0.004)	0.001 (0.004)	**0.0007**	18
denovo15071	*Prevotella copri*	0.617 (0.31)	0.536 (0.29)	0.562 (0.29)	0.203 (0.31)	0.7824	28
denovo15213	*Acidaminococcus*	0.184 (0.03)	0.168 (0.03)	0.032 (0.03)	0.072 (0.03)	**0.0058**	29
denovo15496	*Prevotella*	3.809 (1.01)	1.776 (1.00)	2.138 (1.00)	1.060 (1.01)	0.4188	28
denovo15690	Unclassified Veillonellaceae	0.025 (0.009)	0.042 (0.009)	0.007 (0.009)	0.001 (0.009)	**0.0094**	17
denovo15726	Unclassified Paraprevotellaceae	0.066 (0.01)	0.027 (0.01)	0.008 (0.01)	0.009 (0.01)	**0.0098**	22
denovo16088	*Prevotella*	0.367 (0.08)	0.065 (0.08)	0.070 (0.08)	0.018 (0.08)	**0.0269**	28
denovo17011	Unclassified Veillonellaceae	0.242 (0.05)	0.316 (0.05)	0.108 (0.05)	0.128 (0.05)	**0.0327**	31

^1^Data is shown as LSMeans with standard errors in parentheses.

^2^ *n* = 8 among groups.

^3^Bold *P*-values indicate groups that differ (*P*<0.05).

^4^Percentage of total sequences for steers with nondetectable OTUs were treated as 0.001%.

### Effect of Gain and Intake

The association of the microbial populations with ADG and ADFI were analyzed similarly to the aforementioned feed efficiency data in order to determine whether microbial populations differed by low vs. high ADG, low vs. high ADFI, or their interaction. This was conducted to determine the association of microbial population factors contributing to feed efficiency. Tables 4 and 5 list the significant relative abundances of taxa and OTUs between ADG and ADFI groups (*P*<0.01). As expected, many of the same taxa and OTUs listed in the feed efficiency data were present, with the majority of the populations associating with ADG. Gain-associated taxa included the phylum Lentisphaerae (*P* = 0.00688), families Veillonellaceae (*P* = 0.00184) and Lachnospiraceae (*P =* 0.00214), and the genera *Dialister* (*P =* 0.00005) and *Acidaminococcus* (*P* = 0.00460). Although many gain-associated OTUs were identified, only three OTUs were intake-associated, which included order Clostridiales (*P* = 0.0097) and families Coriobacteriaceae (*P =* 0.0006) and Veillonellaceae (*P =* 0.0024).

**Table 4:** Relative abundance of taxa within ADG and ADFI.

	Phenotype^1^ ^,^ ^2^					
Classification	ADG_High_	ADG_Low_	ADFI_High_	ADFI_Low_	Effect	*P*-Value^3^
*Dialister*	3.388 (0.377)	0.783 (0.377)	1.944 (0.391)	2.227 (0.364)	gain	0.00005
Unclassified Family Veillonellaceae	3.542 (0.406)	1.546 (0.406)	2.125 (0.421)	2.963 (0.392)	gain	0.00184
Unclassified Family Lachnospiraceae	4.98 (0.463)	2.74 (0.463)	3.961 (0.48)	3.759 (0.447)	gain	0.00214
*Acidaminococcus*	0.288 (0.048)	0.076 (0.048)	0.212 (0.05)	0.152 (0.047)	gain	0.00460
Phylum Lentisphaerae	0.01 (0.015)	0.071 (0.015)	0.051 (0.015)	0.03 (0.014)	gain	0.00688
Unclassified Family Victivallaceae	0.01 (0.015)	0.07 (0.015)	0.051 (0.015)	0.029 (0.014)	gain	0.00789
Family Erysipelotrichaceae; Genus *RFN20*	0.368 (0.073)	0.292 (0.073)	0.281 (0.076)	0.379 (0.071)	gain*intake	0.00366

^1^ Data is shown as LSMeans with standard errors in parentheses.

^2^ *n* = 8 among groups.

^3^ *P*-values indicate groups that differ (*P*<0.01).

**Table 5:** Relative abundance of OTUs within ADG and ADFI.

		Phenotype^1^ ^,^ ^2^					
OTU ID	Classification	ADG_High_	ADG_Low_	ADFI_High_	ADFI_Low_	Effect	*P*-Value^3^
denovo4476	Unclassified Order Bacteroidales	0.013 (0.005)	0.041 (0.005)	0.035 (0.005)	0.019 (0.005)	gain	0.0007
denovo15213	*Acidaminococcus*	0.176 (0.023)	0.053 (0.023)	0.128 (0.024)	0.1 (0.022)	gain	0.0007
denovo3724	*Dialister*	1.321 (0.196)	0.269 (0.196)	0.64 (0.203)	0.95 (0.189)	gain	0.0008
denovo5361	*Bulleidia*	0.001 (0.002)	0.01 (0.002)	0.006 (0.002)	0.005 (0.002)	gain	0.0011
denovo12680	*Succiniclasticum*	0.417 (0.078)	0.039 (0.078)	0.264 (0.081)	0.192 (0.075)	gain	0.0021
denovo15690	Unclassified Family Veillonellaceae	0.034 (0.006)	0.004 (0.006)	0.013 (0.006)	0.025 (0.006)	gain	0.0022
denovo12086	*Prevotella*	0.325 (0.054)	0.067 (0.054)	0.101 (0.056)	0.292 (0.052)	gain	0.0022
denovo8962	*Prevotella*	0.224 (0.034)	0.062 (0.034)	0.169 (0.035)	0.117 (0.033)	gain	0.0026
denovo693	Unclassified Family Veillonellaceae	0.028 (0.005)	0.004 (0.005)	0.018 (0.005)	0.014 (0.005)	gain	0.0030
denovo10455	Unclassified Order Streptophyta	0.008 (0.001)	0.002 (0.001)	0.005 (0.001)	0.005 (0.001)	gain	0.0032
denovo4580	*Selenomonas*	0.1 (0.019)	0.011 (0.019)	0.072 (0.02)	0.038 (0.019)	gain	0.0033
denovo15726	UnclassifiedFamily Paraprevotellaceae	0.047 (0.009)	0.008 (0.009)	0.038 (0.009)	0.017 (0.009)	gain	0.0047
denovo10250	*Prevotella*	0.009 (0.002)	0.002 (0.002)	0.006 (0.002)	0.006 (0.002)	gain	0.0058
denovo10446	UnclassifiedFamily Veillonellaceae	0.173 (0.026)	0.064 (0.026)	0.102 (0.027)	0.135 (0.025)	gain	0.0059
denovo9246	*Dialister*	0.636 (0.124)	0.114 (0.124)	0.377 (0.128)	0.372 (0.119)	gain	0.0062
denovo13008	Unclassified Family Veillonellaceae	0.082 (0.017)	0.012 (0.017)	0.038 (0.017)	0.056 (0.016)	gain	0.0064
denovo17011	Unclassified Family Veillonellaceae	0.279 (0.038)	0.119 (0.038)	0.185 (0.04)	0.213 (0.037)	gain	0.0064
denovo14434	Unclassified Family Veillonellaceae	0.018 (0.003)	0.005 (0.003)	0.004 (0.003)	0.019 (0.003)	gain	0.0071
denovo10316	Unclassified Family Veillonellaceae	0.062 (0.014)	0.007 (0.014)	0.028 (0.014)	0.041 (0.013)	gain	0.0079
denovo1046	Unclassified Family Lachnospiraceae	1.627 (0.294)	0.437 (0.294)	0.977 (0.305)	1.087 (0.284)	gain	0.0084
denovo9974	*Butyrivibrio*	0.086 (0.02)	0.009 (0.02)	0.036 (0.02)	0.059 (0.019)	gain	0.0098
denovo12086	*Prevotella*	0.325 (0.054)	0.067 (0.054)	0.101 (0.056)	0.292 (0.052)	gain*intake	0.0097
denovo4857	Unclassified Family Coriobacteriaceae	0.01 (0.002)	0.009 (0.002)	0.015 (0.002)	0.004 (0.002)	intake	0.0006
denovo14434	Unclassified Family Veillonellaceae	0.018 (0.003)	0.005 (0.003)	0.004 (0.003)	0.019 (0.003)	intake	0.0024
denovo621	Unclassified Order Clostridiales	0.007 (0.002)	0.008 (0.002)	0.012 (0.002)	0.004 (0.002)	intake	0.0097

^1^ Data is shown as LSMeans with standard errors in parentheses.

^2^ *n* = 8 among groups.

^3^ *P*-values indicate groups that differ (*P*<0.01).

## Discussion

In this study, the V1–V3 *rrs* region was chosen to analyze the microbial diversity within the rumen of steers. A myriad of studies report microbial ecology data utilizing 16S variable regions, and due to read lengths available for the most common next generation sequencing platforms, a subset of variable segments are targeted, rather than the entire length of the gene. To this extent, data regarding each specific ecological community can be confounded and biased based upon the variable region(s) selected [24–26]. To that end, careful consideration was taken to best represent the microbial communities in the rumen and lower GI. The region spanning the V1–V3 regions was selected for the present study, based on analysis of ruminal and GI studies to maximize ability to compare across this and previous studies.

The study was able to recover 98 to 99% of all OTUs calculated at 0.03 dissimilarity, as determined by Good's coverage estimator. Rarified samples to 25,000 sequences/sample demonstrated adequate depth, as demonstrated in previous studies [27]. However, greater coverage may be warranted in certain cases, as noted by an *in silico* study in which the microbial diversity in the rumen was compared against all curated 16S rRNA gene sequences in the RDP database [28]. In this comparison, coverage up to 80,000 reads/sample was estimated to be minimal in order to adequately capture the OTUs at species level (0.03 phylogenetic distance). However, the percent coverage metric used for this conclusion was not the same metric used in this study or in that of Jami and Mizrahi (2012) [27], and thus the requisite number of reads cannot be directly compared.

Greater sequencing depth and number of animals per group in the present study, compared to an earlier attempt to correlate microbiome composition with feed efficiency phenotypes [29], allowed numerous taxonomic and OTU classification links to be established in our experiment. Although the present study had greater observed OTUs, more in keeping with other estimates of rumen microbial richness, diversity analyses of the OTUs identified across all samples and groups, revealed no differences in observed OTUs, richness (Chao1), or diversity (Shannon; Table 1). However, the data cannot be accurately contrasted to the previous study, due to analysis of different 16S variable regions, as noted previously. Moreover, finer comparisons between studies are impeded by the inherent variability observed in ruminal microbial community datasets. Within-sample variation was minimal based on technical replicates from the same rumen fluid sample, at approximately 0.2% of taxa representing ≥1% of the reads, while animal-to-animal variation in the ruminal bacterial community within feed efficiency group was as large as 3.0% in the current study. It has previously been reported that variation between rumen metagenome profiles of different cattle is greater than of rumen metagenome profiles from repeated samples on the same animal [30]. We had hypothesized that increases in sample size could overcome such variation within the microbial communities in the rumen, but this was not the case. In addition to the diversity analyses, the weighted and unweighted UniFrac PCoA examining the phylogenetic divergence between the OTUs did not separate or cluster, further supporting the similarities between the microbial communities within each group (Fig 2).

It is possible that the diversity analyses may not be the correct level at which to discover microbiome variability impacts on feed efficiency phenotypes. The lack of differences observed between feed efficiency groups at the level of diversity analyses may simply indicate that the important variation in microbial communities lie at a finer resolution. For example, variation among or within specific taxa and OTUs provided by partial 16S sequencing may be more informative rather than the number and diversity of taxa and OTUs. Nevertheless, the lack of a noted change in diversity (Shannon) between the feed efficiency phenotypes was unexpected, given that obesity or extremes in BW have been associated with changes in the microbial ecology and diversity of intestinal microbial populations in humans and mice [6–7]. In contrast, other studies have shown that host specificity of the ruminal bacterial community may account for some of the similarities observed in microbial diversity, as well as contribute to the substantial animal-to-animal variation [31], which may play an important role in this study.

There is limited research examining the effect of the rumen microbiome on feed efficiency, especially with regard to the phenotypes delineated in our model (Fig 1). As noted previously, there were no observable differences between the groups when evaluating OTU alpha and beta diversity (Table 1, Fig 2). However, the relative taxonomic abundance may still be affected, which was the case when examining the relative taxonomic profiles at the phylum and sub-phylum levels (Table 2). Many of the changes were identified within the phylum Firmicutes, which included families Lachnospiraceae and Veillonellaceae, and genera *Acidaminococcus*, *Dialister*, and *Anaerovibrio*. Interestingly, increases in the abundance of Firmicutes, altering the Firmicutes-to-Bacteroidetes ratio, have been shown to affect energy harvesting and were correlated with increases of fat [23]. Our study found that increases in Firmicutes were associated with animals of greater ADG (Table 2), and many of the genera belonging to Firmicutes were correlated with high ADG (Table 5). The great abundance of Firmicutes within the rumen suggests that these shifts may play a significant role in affecting feed efficiency.

The rumen content from the four feed efficiency groups revealed significant differences in the relative abundance of specific taxa (Table 2). Many members of the family Lachnospiraceae have cellulolytic activity and are closely associated with other cellulose-degrading bacteria [32, 33]. *Acidaminococcus* are amino acid-fermenting bacteria [34] and are also related to the butyrate producing *Butyrivibri*o, which was also detected from changes in feed efficiency (Table 3). In addition, *Anaerovibrio* has been associated with succinate and propionate production, as well as lipid hydrolysis and metabolism in ruminant animals [35]. Increases in *Dialister* populations have been associated with hyposalivation [36], which may play a role in altering the buffering capacity of the rumen and fluid turnover. The phylum Lentisphaerae was also associated with changes in feed efficiency, and differences in Lentisphaerae populations have been observed in the rumen during subacute ruminal acidosis [37]. Additionally, lipopolysaccharide challenge has been shown to linearly decrease Bacteroidetes and, in part, Lentisphaerae populations possibly leading to a decrease in fermentative activity [38]. However, little data has been presented on their role of feed efficiency in the rumen, and their significance and ruminal residence remains to be determined.

Differences in many OTU abundances were also detected, specifically in the genera *Succiniclasticum*, *Lactobacillus*, *Ruminococcus*, many *Prevotella*, and the family Veillonellaceae (Table 3). *Succiniclasticum* was detected at greatest abundance in the ADG_Low_-ADFI_High_ group, and its relevance lies in being part of order Selenomonadales and specializing in fermenting succinate and converting it to propionate [39]. Veillonellaceae are also known to produce propionate as a major fermentation product [40]. The observation that *Succiniclasticum* were at higher levels in the ADG_Low_-ADFI_High_ group, while Veillonellaceae were decreased in abundance within the same group, may indicate resource competition among the organisms. A higher abundance of propionate-producing bacteria may divert H_2_ away from methanogenesis reducing methane emissions [8]. *Ruminococci* are known to be cellulolytic as well as active in acetate, formate, and hydrogen production [41]. Finally, changes in the abundances of *Lactobacillus* were not completely unexpected, as these organisms are high when concentrate diets are fed [42]. It must be noted however, that although the changes in taxa and OTUs and their putative functions in the rumen can be correlated with the observed differences in the phenotypes, it is not clear whether changes in the microbiome are contributing to differences in feed efficiency or host factors are driving changes in the microbiome.

Trends were observable within the relative taxonomic abundance of the efficiency groups, but did not follow previously observable taxonomic affects. For instance, *Prevotella* abundance has been shown to be greater in inefficient animals [43]. Yet, this was not adequately demonstrated in the feed efficiency microbial profiles. *Prevotella* was in abundance at 57.2, 45.4, 49.6, and 47.6% for the ADG_High_-ADFI_High_, ADG_High_-ADFI_Low_, ADG_Low_-ADFI_Low_, and ADG_Low_-ADFI_High_ groups, respectively (Table 2). Our study did however parallel the accepted abundance of *Prevotella* seen in other studies regarding ruminants at 42% to 60% of the bacterial 16S rRNA gene sequences [44,45]. *Prevotella* has been reported as the most abundant ruminal genus, which is in agreement with our study [45].

The unassigned taxa accounted for approximately 9% of the reads. This data has yet to be resolved, but may be important in understanding the entire ruminal microbiome. Species associated with these unassigned taxa may play a yet to be established, important role in feed efficiency. A community-based microbial database would be the most promising option in determining an accurate microbiome. In such cases, not only would database hits increase, limiting unassigned taxa, but accuracy in reporting microbial changes and variability from altered parameters would enhance analysis as well. A current study demonstrated the importance of establishing such a system [30]. The authors developed a reference metagenome to compare rumen metagenomic profiles for individual cattle. When the reads from the study were aligned to a rumen metagenome reference, the rumen metagenome profiles were repeatable (*P* < 0.00001) within sample regardless of location of sampling rumen fluid. In addition, repeatability was approximately 9%, however because of the small number of cattle used in the study, there was a higher standard error. They were also able to detect variability between samples from different cattle, demonstrating the effectiveness and precision of utilizing a community-specific reference microbial database.

Changes in the microbial populations were also evident when examining ADG and ADFI individually or their interaction. Interestingly, the significant changes in microbial abundances were primarily affected by ADG, with many of the same organisms identified in the feed efficiency study. These data permit further evaluation of the microbial abundances, implicating not only the eventual microbial effect in that of feed efficiency, but also its components, relating information on how population shifts affect feed efficiency.

The majority of taxa and OTUs indentified as associating with changes in feed efficiency in this study were related to cellulolytic, fermentative, and metabolic activities in the cattle rumen. Alterations in their abundances therefore correlate to changes in observed feed efficiency phenotypes, and necessitate further study to examine, alter, and optimize microbial populations for improved feed efficiency.

Although others have demonstrated differences in feed efficiency using community-based PCR-denaturing gradient gel electrophoresis (DGGE) and targeted quantitative real-time PCR analysis [46], this was one of the first studies to directly examine the effect of rumen microbial communities and abundances on feed efficiency, ADFI, and ADG of cattle at the 16S level utilizing next-generation sequencing technologies. Additionally, the study had sufficient power and depth, in contrast to previous research. Although, differences in microbiomes among feed efficiency phenotype class could not be detected at the phylum or genus level by PCoA, diversity indices, or observed OTU count, relative taxonomic abundance and OTU classifications indicated many significant changes in microbial populations as a function of feed efficiency. These populations are also responsible for many of the transformations known to occur in the rumen. However, other analyses may better determine microbial effects on feed efficiency. A metagenomic approach could allow for greater resolution analyses, for example at the level of cellulase genes present and/or expressed [47]. Finally, analyzing the microbial communities throughout the lower GI (jejunum, cecum, and colon) as a function of feed efficiency should also aid in understanding the complete relationship between microbial communities and feed efficient cattle.
